# A web-survey assessed attitudes toward evidence-based practice among psychotherapists in Austria

**DOI:** 10.1038/s41598-022-13266-2

**Published:** 2022-06-07

**Authors:** B. Nussbaumer-Streit, A. Jesser, E. Humer, A. Barke, B. K. Doering, B. Haid, W. Schimböck, A. Reisinger, M. Gasser, H. Eichberger-Heckmann, P. Stippl, G. Gartlehner, C. Pieh, T. Probst

**Affiliations:** 1 Department for Evidence-Based Medicine and Evaluation, University for Continuing Education Krems, Dr.-Karl-Dorrek Strasse 30, 3500 Krems a.d. Donau, Austria; 2 Department for Psychotherapy and Biopsychosocial Health, University for Continuing Education Krems, 3500 Krems, Austria; 3 Clinical and Biological Psychology, Catholic University of Eichstätt-Ingolstadt, 85072 Eichstätt, Germany; 4 Brandenburg Medical School Theodor Fontane, Neuruppin, Germany; 5 Austrian Federal Association for Psychotherapy, 1030 Vienna, Austria; 6 ABILE-Viktor Frankl Education Austria, 3390 Melk, Austria; 7 PROGES, 4020 Linz, Austria; 8 RTI International, 3400 Cornwallis Rd, Research Triangle Park, NC USA

**Keywords:** Psychology, Health care

## Abstract

Evidence-based practice (EBP) means integrating the best available scientific evidence with clinical experience and patient values. Although perceived as important by many psychotherapists, there still seems to be reluctance to use empirically supported therapies in clinical practice. We aimed to assess the attitudes of psychotherapists in Austria toward EBP in psychotherapy as well as factors influencing the implementation of EBP. We conducted an online survey. To investigate attitudes toward EBP, we used two subscales (“Limitations” and “Balance”) of a translated and validated short version of the Evidence-Based Practice Attitude Scale-36 (EBPAS-36). Participants provided perceived barriers and facilitators as answers to open-ended questions. We analyzed the responses mainly using descriptive statistics. Open answers were analyzed using a thematic analysis. In total, 238 psychotherapists completed our survey (mean age 51.0 years, standard deviation [SD] = 9.9, 76.9% female). Psychotherapists scored on average 2.62 (SD = 0.89) on the reversed EBPAS-36 subscale “Limitations,” indicating that the majority do not perceive EBP as limiting their practice as psychotherapists. They scored 1.43 (SD = 0.69) on the reversed EBPAS-36 subscale “Balance,” indicating that psychotherapists on average put a higher value on the art of psychotherapy than on evidence-based approaches. Organizational factors such as lack of time and access to research studies as well as negative attitudes toward research and a lack of skills and knowledge kept respondents from implementing EBP. Our study highlights that EBP is still not very popular within the psychotherapy community in Austria. The academization of psychotherapy training might change this in the future.

## Introduction

Evidence-based practice (EBP) means integrating the best available scientific evidence with clinical experience and patients’ values or preferences^1^. This approach has been internationally widely implemented in the medical field medicine since its introduction in the 1990s. In other health professions such as psychotherapy, EBP is still quite a novel concept^2^. Although EBP has many benefits, and research has shown that implementing EBP can improve clinical outcomes in psychotherapy compared to usual care^3–5^, many psychotherapists remain skeptical whether it is useful in the field of psychotherapy^2^.

Surveys have shown that, in general, most psychotherapists think that evidence from research studies is relevant to their practice^6,7^. However, many perceive such evidence as less influential than other sources of information, such as clinical experience or advice from colleagues^8^. Studies have also reported low use of empirically supported therapies in clinical practice^9–14^. Cook et al. summarize a range of potential barriers to implementing EBP in clinical practice. One major barrier was a negative attitude toward controlled research studies or evidence-based manuals, which are perceived as lacking applicability to real-world settings as well as providing limited flexibility to tailor therapies to individual patients^15^. Gaudiano et al. showed that, in particular, psychotherapists who tend to rely on their intuition and perceive psychotherapy as more of an art than a science refuse to implement an evidence-based approach in practice^16^. Other barriers were lack of time, lack of training, institutional constraints (e.g., lack of access to resources or reimbursement), or that an evidence-based approach was not in accordance with the therapeutic philosophy already in place^15^.

Barriers to implementing EBP in psychotherapy varied with clinicians’ characteristics. Practitioners with more professional education had more favorable attitudes toward EBP than those with less education^17^. Resistance was also more prominent in older practitioners who received their diplomas before EBP entered the field of psychotherapy than in younger ones^18^.

Austria has a long tradition of psychotherapy going back to the 1920s and a wide range of established psychotherapeutic schools of thought. Psychotherapists can choose from 23 accredited psychotherapeutic schools in Austria^19^, which can be summarized into four psychotherapeutic orientations: psychodynamic, humanistic, systemic, and behavioral. Although educational programs at some training institutions have integrated EBP into their curriculum within the last few years, many psychotherapeutic schools accredited in Austria still do not fulfill the criteria to be regarded as evidence based.

The aim of our study was therefore to assess the attitudes of psychotherapists in Austria toward EBP in psychotherapy. Specifically, we wanted to answer the following research questions:

- Question 1: What are the attitudes of Austrian psychotherapists toward EBP?
- Question 2: Do psychotherapists’ attitudes toward EBP vary by age, gender, psychotherapeutic orientation, years of experience, professional status (licensed or in training), setting, and patient group (working with adults only vs. with children, adolescents, and adults)?
- Question 3: How much time do psychotherapists spend informing themselves about the latest research in their specialty, and what information sources do they use?
- Question 4: What factors act as barriers when working according to EBP standards, and what could be facilitators?

## Methods

To answer our research questions, we conducted an online survey comprising 40 items in total using REDCap (Vanderbilt University, Tennessee, United States)^19,20^. The current study was part of a larger study previously published by Humer et al. examining the provision of psychotherapy in Austria one year after the onset of the COVID-19 pandemic^21^. We received approval from the ethical review board of the University for Continuing Education Krems (EK GZ 23/2018–2021); the study was not registered elsewhere. The ethical review board of the University for Continuing Education Krems provided guidelines on study conduct as stated in Section IX § 2 of the Statutes of the University for Continuing Education Krems and we adhered to them. Throughout the manuscript, we adhere to the CHEcklist for Reporting Results of Internet Surveys (CHERRIS) guidelines for online surveys^22^.

### Sampling frame

Psychotherapist is a licensed profession in Austria. Psychotherapy training is offered by institutions accredited by the Austrian Federal Ministry of Social Affairs, Health, Care and Consumer Protection. An institution can be accredited for one or multiple therapeutic approaches. Training is regulated by the Austrian psychotherapy law and comprises two parts: first 765 h of lessons in theory and 550 h of practical training, second 300 advanced lessons in theory and 1600 h of practical training. Persons who are at least 28 years old and who have completed the required training are allowed to work as psychotherapists in Austria. Most psychotherapist, in addition to their psychotherapy training have training in another professional field such as psychology, medicine, or social work. However, an additional profession is not a prerequisite. The Austrian Federal Association for Psychotherapy (Vienna, Austria) supported recruitment for this study by sending their members an information e-mail. In addition, all licensed Austrian psychotherapists who are registered with the Austrian Federal Ministry of Social Affairs, Health, Care and Consumer Protection as psychotherapists and who had provided a valid e-mail address (about 6,000 psychotherapists) were invited to participate in the survey.

### Survey method

Assessing the attitudes toward EBP was part of a larger study for which we developed a German-language questionnaire that comprised the following thematic areas: provision of psychotherapy in face-to-face contact, via telephone or internet, self-assessment bias, and attitudes toward EBP. First, we asked about age, gender, patient group treated (adults only vs. children, adolescents, and adults), experience (licensed vs. in training), setting (private, outpatient, inpatient), and therapeutic orientation (only the four psychotherapeutic orientations: psychodynamic, humanistic, systemic, behavioral). Second, to investigate attitudes toward EBP, we used two subscales (“Limitations” and “Balance”) of a translated and validated short version of the Evidence-Based Practice Attitude Scale-36 (EBPAS-36)^23,24^. The “Limitations*”* subscale assessed whether EBP is perceived as useful in working with clients or as too narrowly focused or not individualized enough. The “Balance” subscale assessed whether psychotherapy is perceived as more of an art than a science, or whether it is both, and whether psychotherapists perceive their competence as a therapist as more important than a particular approach. We also asked psychotherapists about the proportion of time they spend informing themselves on the current evidence in their field. Finally, we asked three open-ended questions about the sources used for finding research evidence as well as the perceived barriers and facilitators that influence the individual implementation of EBP.

Before starting the survey, two team members pretested its usability and technical functionality. We conducted the survey between February 16, 2021 and April 2, 2021 by sending out one general link. We included only completed surveys in our analysis to avoid multiple entries from the same person (e.g., by multiple tries to complete the survey). The data collection was anonymous, and the data was stored in REDCap because this allowed us to store data on a local campus server and to be in compliance with European data protection legislation.

### Data analysis

We analyzed the responses mainly using descriptive statistics. For the outcome “evidence-based practice attitude,” we calculated the mean scores and standard deviations (SDs) of the “Limitations” and “Balance” subscales of the EBPAS-36. These subscales comprise three items; each can be rated from 0 to 4 points. A higher score on the “Limitations” and “Balances” subscales indicates a more negative attitude toward EBP. Considering all items in the EBPAS-36, most are worded in a way where a higher total score indicates a more positive attitude toward the adoption of EBP^23^. Therefore, users of the scale reverse the ratings of the “Limitations” and “Balance” subscales to compute an overall score. Although we did not calculate an overall EBPAS-36 score, we reversed the ratings of the two subscales to make our results comparable with the results from other studies. To assess the mean proportion of time psychotherapists spend on informing themselves about the latest research in their specialty, we calculated the mean and SD. We presented the sources searched by psychotherapists as proportions. To explore differences in attitudes between gender, patient group (working with adults only vs. with children, adolescents, and adults), and professional status (licensed vs. in training), we used independent t-tests, with a significance level of *p* ≤ 0.05. To explore the mean differences in attitudes among multiple groups (psychotherapeutic orientation and setting), we calculated a one-factorial analysis of variance (ANOVA) after Kruskal–Wallis and corrected for multiple testing within this comparison using the Bonferroni correction. Due to the exploratory nature of the analysis, we did not correct for multiple comparisons across the different tests. We calculated Pearson coefficients to assess correlations between age as well as years of experience and the two EBPAS subscales “Limitations” and “Balance.” We completed all calculations in IBM SPSS Statistics Version 26 for Windows (Chicago, IL, USA^25^).

Psychotherapists were asked to name one or more factors that hinder or facilitate their implementation of EBP. We analyzed the answers using an inductive thematic analysis^26^. We assigned initial codes to the responses and combined similar codes into subthemes and overarching themes. One researcher performed the initial coding. Two researchers independently combined similar codes into subthemes and overarching themes. The interim results were discussed and revised with another researcher to corroborate the findings. We calculated the interrater reliability of the two researchers when grouping the codes into themes.

### Ethical approval and consent to participate

We received approval from the ethical review board of the University for Continuing Education Krems (EK GZ 23/2018–2021). Participants were informed about the aim of the study and were guaranteed that all given information will be presented in an anonymized way. Participation in the survey was voluntary, and we did not offer any incentives for completion. To access the survey, participants had to provide informed consent and agree to the data protection declaration (electronic informed consent).

## Results

### Respondents

In total, there are more than 10,579 licensed psychotherapists in Austria (July 2021), and 238 completed our survey (17 were not yet licensed but in training under supervision). Their mean age was 51.0 years (SD = 9.9) and three-quarters were female. The majority (92.9%) were registered psychotherapists with a median of 11 years of experience (1–36 years); the other 7.1% were psychotherapists in training. Three-quarters worked in a single workplace, the others in two (24%) or even three in parallel (1%). Most psychotherapists worked in private practice (97.9%), 20.2% in an outpatient clinic setting, and 8.4% in an inpatient setting. Half of the participants worked exclusively with adults, and the other half with adults, children, and adolescents (see Table 1).

**Table 1:** Characteristics of the participants.

Characteristics	Number of participants (%)
**Gender (n = 238)**	
Female	183 (76.9)
Male	54 (22.7)
Diverse	1 (0.4)
**Age (n = 238)**	
< 30	3 (1.3)
31–40	35 (14.7)
41–50	71 (29.8)
51–60	94 (39.5)
> 60	35 (14.7)
**Professional status (n = 238)**	
Licensed psychotherapist	221 (92.9)
Psychotherapist in training	17 (7.1)
**Years of experience (n = 221)**	
< 5	49 (22.2)
5–10	59 (26.7)
11–20	59 (26.7)
> 20	54 (24.4)
**Workplace (n = 238, multiple answers possible)**	
Private practice	233 (97.9)
Outpatient setting	48 (20.2)
Inpatient setting	20 (8.4)
**Work in one or multiple workplaces**	
One setting	178 (74.8)
Two settings	57 (23.9)
Three settings	3 (1.3)
**Patient group (n = 238)**	
Adults	119 (50.0)
Adults, children, adolescents	119 (50.0)
**Psychotherapeutic orientation (n = 229)**	
Psychodynamic	39 (16.4)
Humanistic	121 (50.8)
Systemic	48 (20.2)
Behavioral*	21 (8.8)

The sample size for psychotherapeutic orientation is smaller, since we excluded those with multiple answers (n = 9) to be able to calculate independent comparisons between the orientations. The sample size for “years of experience” is smaller, since we excluded psychotherapists in training (n = 17).

Abbreviations: n = number of respondents.

* Behavioral therapy in Austria comprises all interventions with roots in behavioral therapy including cognitive-behavioral therapy and behavioral therapies of the third wave such as mindfulness-based cognitive therapy.

### Attitudes toward EBP among psychotherapists

Psychotherapists scored on average 2.62 (SD = 0.89) on the reversed EBPAS-36 subscale “Limitations,” indicating that the majority do not perceive EBP as limiting their practice as psychotherapists. Nearly half of the respondents consider EBP as useful for clients with multiple problems. Two-thirds of psychotherapists think EBP allows for individualized treatment. More than half of the participants stated that EBP is not, or is only to a slight extent, too narrowly focused (see Table 2).

**Table 2:** EBPAS-36 “Limitations” subscale.

“Limitations” subscale	M (SD), sample size, percentage of responses
**EBP is not useful for clients with multiple problems**	2.39* (1.28), n = 238
NOT at all (0)	26.5%
slight extent (1)	21.0%
Moderate extent (2)	25.6%
Great extent (3)	18.5%
Very great extent (4)	8.4%
**EBP is not individualized treatment**	2.97* (1.05), n = 238
Not at all (0)	37.8%
Slight extent (1)	33.6%
Moderate extent (2)	18.5%
Great extent (3)	7.6%
Very great extent (4)	2.5%
**EBP is too narrowly focused**	2.51* (1.09), n = 238
Not at all (0)	20.2%
Slight extent (1)	33.2%
Moderate extent (2)	28.2%
Great extent (3)	14.3%
Very great extent (4)	4.2%

*Reversed ratings; a higher value indicates a more positive attitude toward EBP.

Abbreviations: EBP = evidence-based practice, EBPAS-36 = Evidence-Based Practice Attitude Scale-36, M = mean, n = number of respondents, SD = standard deviation.

On average, psychotherapists scored 1.43 (SD = 0.69) on the reversed EBPAS-36 subscale “Balance,” indicating that psychotherapists on average put a higher value on the art of psychotherapy than on evidence-based approaches. Most respondents think their competence as a therapist is more important than a particular approach. However, nearly all agreed, from a moderate to (very) great extent, that therapy is both an art and a science (see Table 3).

**Table 3:** EBPAS-36 “Balance” subscale.

“Balance” subscale	M (SD), n, proportion of responses*
**A positive outcome in therapy is an art more than a science**	2.34* (1.02), n = 238
Not at all (0)	15.5%
Slight extent (1)	28.6%
Moderate extent (2)	38.2%
Great extent (3)	14.3%
Very great extent (4)	3.4%
**Therapy is both an art and a science**	0.86* (1.04), n = 238
Not at all (0)	2.9%
Slight extent (1)	3.8%
Moderate extent (2)	18.5%
Great extent (3)	25.6%
Very great extent (4)	49.2%
**My overall competence as a therapist is more important than a particular approach**	1.05* (0.96), n = 238
Not at all (0)	1.7%
Slight extent (1)	6.3%
Moderate extent (2)	18.9%
Great extent (3)	41.2%
Very great extent (4)	31.9%

*Reversed ratings; a higher value indicates a more positive attitude toward EBP.

Abbreviations: EBP = evidence-based practice, EBPAS-36 = Evidence-Based Practice Attitude Scale-36, M = mean, n = number of respondents, SD = standard deviation.

We explored whether gender, age, professional status, patient group, therapeutic orientation, and amount of experience made any difference in attitudes toward EBP (Table 4). The only two statistically significant differences we found on the EBPAS-36 “Limitations” subscale indicated that behavioral psychotherapists have a more positive attitude toward EBP than humanistic psychotherapists. However, sample sizes in the subgroups were very small, so the results must be interpreted with caution. Age was negatively correlated with the EBPAS-36 “Limitations” subscale, indicating that older psychotherapist had a more negative attitude toward EBP than younger psychotherapists. We saw a slightly smaller correlation (without statistical significance) indicating a more negative attitude toward EBP with more years of experience.

**Table 4:** EBPAS-36 “Limitations” and “Balance” subscales across subgroups.

	EBPAS-36 “Limitations”		EBPAS-36 “Balance”	
Characteristics	M, SD	MD, *p*-value	M, SD	MD, *p*-value
**Gender**				
Female (n = 183)	2.64* (0.87)	0.10, *p* = 0.449	1.45* (0.70)	0.09, *p* = 0.413
Male (n = 54)	2.54* (0.93)		1.36* (0.65)	
**Professional status**				
Licensed (n = 221)	2.60* (0.87)	− 0.26, *p* = 0.242	1.43* (0.69)	0.04, *p* = 0.815
In training (n = 17)	2.86* (1.09)		1.39* (0.64)	
**Patient group**				
Adults (n = 119)	2.59* (0.92)	− 0.06, *p* = 0.609	1.39* (0.68)	− 0.07, *p* = 0.433
Adults, children, adolescents (n = 119)	2.65* (0.85)		1.46* (0.69)	
**Psychotherapeutic orientation**		Kruskal–Wallis Test: *p* = 0.008**		Kruskal–Wallis Test: *p* = 0.108
Psychodynamic (n = 39)	2.65* (0.89)	P vs. H: 0.16, *p* = 1.000 P vs. S: − 0.02, *p* = 1.000 P vs. B: − 0.57, *p* = 0.078 H vs. S: − 0.18, *p* = 1.000 H vs. B: − 0.73, *p* = 0.004*** S vs. B: − 0.55, *p* = 0.110	1.30* (0.67)	No direct comparisons because the overall test for this item showed no statistically significant difference across groups
Humanistic (n = 121)	2.49* (0.87)		1.42* (0.64)	
Systemic (n = 48)	2.67* (0.90)		1.40* (0.74)	
Behavioral (n = 21)	3.22* (0.75)		1.76* (0.84)	

	Pearson correlation	*p*-value	Pearson correlation	*p*-value
**Age**	− 0.159	*p* = 0.014**	− 0.103	*p* = 0.112
Years of experience****	− 0.111	*p* = 0.100	− 0.079	*p* = 0.241

* Reversed ratings; a higher value indicates a more positive attitude toward EBP.

** = Statistically significant difference.

*** = Statistically significant difference (post-hoc test, corrected for multiple testing).

**** = Psychotherapists in training (n = 17) excluded.

Abbreviations: B = Behavioral, EBP = evidence-based practice, H = Humanistic, M = mean, MD = mean difference, *p* = *p*-value, P = Psychodynamic, S = Systemic, SD = standard deviation.

We decided not to test for differences between psychotherapists working in different settings, because the overwhelming majority (97.9%) worked in private practice, and too few representatives worked solely in outpatient or inpatient clinics.

### Informing about research evidence

On average, psychotherapists stated they spend 16.0% (SD 10.9, n = 238) of their working time informing themselves on the current evidence in their field. The majority of the 238 respondents sought information by exchange with colleagues (97%) followed by education (92%) and attendance of scientific congresses (70%). About 55% subscribe to German scientific journals, 42% read systematic reviews, 36% use guidelines and manuals, and 19% subscribe to international journals. Forty percent also use other sources of information such as theses, libraries, online databases (e.g., PubMed), search engines (e.g., Google or Google Scholar), websites from professional associations, websites from colleagues, YouTube videos, and Wikipedia.

### Barriers and facilitators when working according to EBP standards in psychotherapy

We identified three major barriers. *Organizational factors* such as lack of time and lack of access to research studies as well as *negative attitudes* toward research and a *lack of skills and knowledge* kept respondents from implementing EBP. The interrater reliability of the two coders was 88% (93/106 codings).

We identified seven major facilitators. *Organizational factors* such as enough time, easy access to studies, and support from the team or organization helped psychotherapists implement EBP. Participants also named *external input* such as conferences or trainings as helpful. In addition, *exchange with others*, *good packaging of the evidence*, and *the need to use evidence for work* were considered facilitators of EBP. On an individual level, a *positive attitude* as well as *knowledge and skills* helped practitioners use an EBP approach. The interrater reliability of the two coders was 86% (190/222 codings). Table 5 gives an overview of the identified barriers and facilitators and their subthemes.

**Table 5:** Barriers and facilitators for implementing EBP.

Level	Barriers		Facilitators	
External level	Organizational factors	Lack of time Lack of access	Organizational factors	Enough time Cost-free and easy access to studies Support from the team or organization
			External input	Conferences Training/education Talks, videos Books, journals Newsletters
			Exchange with others	Communication with colleagues Intervision Supervision
			Packaging of evidence	Lay language summaries Practical-oriented (e.g., using a case study format)
			Necessary for work	Evidence relevant for job as a psychotherapist (challenges in practice) Evidence relevant for additional job as lecturer, trainer, researcher, editor
Individual Level	Negative attitude	No need/interest in study results Distrust toward studies Study results not generalizable to individual patients	Positive attitude	Research interest Desire to provide state-of-the art psychotherapy Perceiving research as important for work as a psychotherapist Positive experience using evidence
	Lack of knowledge/skills	Studies lack intelligibility Lack of competence/routine	Knowledge/skills	Routine/experience Knowing where to find and how to interpret evidence

Abbreviations: EBP = evidence-based practice.

## Discussion

To the best of our knowledge, this is the first study assessing attitudes toward EBP, information-seeking behavior, and barriers and facilitators for implementing EBP in psychotherapy in Austria. Although psychotherapists mostly perceive EBP as an important aspect of psychotherapy, they think that their experience and competence is more important than following a specific evidence- or manual-based approach. These findings are in line with the results from other studies that indicate a reluctance to implement an evidence-based approach in clinical practice within the field of psychotherapy^8,9^. However, the belief that therapy is more of an art than a science and that overall competence is more important than science was more prominent in our sample than in other countries such as Germany, Norway, and the United States^23^. A potential explanation could be that in Austria psychotherapists do not need to complete an academic education. Learning how to plan, conduct, or use studies is underrepresented in their education. In addition, not all accredited psychotherapeutic orientations in Austria fulfill the criteria to be evidence-based, while in Germany, for example, only evidence-based psychotherapeutic orientations are accredited and eligible for reimbursement^27^.

Respondents named colleagues as the major source of information, while guidelines and systematic reviews were used by about one-third of respondents. In EBP, expert opinions are, although important, considered to be the lowest level of evidence^28^. Additionally, it remains unclear whether all colleagues are experts in their field. It seems necessary to raise awareness within the psychotherapy community that expert opinion is not enough to implement EBP. Such opinions are subjective and have a high risk of being distorted by confirmation bias—the tendency to search and interpret information in a way in which it supports one’s own beliefs—and, therefore, are not sufficient to foster transparent EBP^29^.

Psychotherapists in Austria reported the same barriers to implementing EBP as international studies^15,16^. Most often, they named lack of time as a barrier. The second aspect might be aligned with the participants’ perceived lack of knowledge and skills and the lack of access to evidence (research results). Providing more trainings and integrating courses into psychotherapy curricula to build capacity in EBP as well as more support from academic institutions (e.g., librarians conducting systematic literature searches, cost-free access to databases) could help overcome these barriers.

On an individual level, some psychotherapists had negative attitudes toward EBP. They stated they distrusted study results and did not see the applicability to their individual patients and, therefore, did not perceive evidence from studies as helpful to their work. Enabling psychotherapists to implement meta-skills such as finding trustworthy sources and critically appraising and interpreting study results could help overcome this distrust. In a study from 2006, the findings showed that resistance to EBP was more prominent in older practitioners who received their diplomas before EBP entered the field of psychotherapy than in younger ones^18^. We could also identify this correlation with age in our sample. However, this could be due to a lack of training in EBP or due to a general lack of experience in searching for information online, not necessarily because of resistance to EBP.

Besides the more obvious facilitators such as more time, easier access to evidence, and better knowledge and skills, we also identified novel facilitators that should be considered in the field of psychotherapy. Participants mentioned that presenting evidence in a more user-friendly way, such as translating research results into lay language or combining them with practical examples, would help them use research results for their individual patient work. Instead of needing to search for evidence (pull approach), receiving evidence via newsletters or congresses (push approach) was also perceived as useful. Producers of evidence should consider this in their dissemination and knowledge translation activities. Further, if colleagues and employers support an EBP approach, this helped individual psychotherapists implement it.

## Limitations

Assessing the attitudes toward EBP was part of a larger study. The complete survey aimed to answer multiple research questions and therefore consisted of 40 items. To make completing the survey feasible, we decided to focus on two subscales of the EBPAS-36 instead of using the entire tool. We chose the two subscales that seemed most important for the Austrian context and believe that the results give important insights into the attitudes toward EBP in Austria.

Another limitation is the uncertainty about how representative our results are. Only a small proportion of eligible psychotherapists participated in the survey, and since we did not draw a random sample, the self-selection of participants might have influenced the sample. While the gender distribution is representative for Austria, the psychiatric orientation is not. In our sample psychotherapists with a humanistic orientation are overrepresented (50% vs. 38%) while those with psychodynamic (16% vs. 26%) is underrepresented^30^.

The survey was conducted during the Corona virus pandemic, which might have had an influence on the perception of evidence-based practice as evidence-based medicine was a prominent topic during COVID-19. It should also be kept in mind that most Austrian psychotherapists changed the treatment format for the first time from face-to-face psychotherapy to tele-psychotherapy (i.e., psychotherapy via telephone or Internet) due to COVID-19 related restrictions^31^. In general, Austrian psychotherapists rated psychotherapeutic interventions such as psychodynamic, humanistic, systemic, cognitive, behavioral interventions as less typical for tele-psychotherapy than for face-to-face psychotherapy during the pandemic^32^. Moreover, differences between therapeutic orientations in behavioural-oriented interventions—which have the most evidence-base in the context of psychotherapy—have been shown to become less prominent in tele-psychotherapy as compared to face-to-face psychotherapy in Austria during the pandemic^32^. In addition, we cannot rule out that participants provided socially desirable answers (e.g., spending 16.0% of their working time informing themselves on the current evidence seems unrealistically high), although we guaranteed full anonymity to all participants.

We focused on Austrian psychotherapists in our sample, compromising the generalizability of our findings to other countries. However, the findings are generally in line with the existing research^15,16^. In particular, the identified barriers are in accordance with those reported in other studies, indicating that there are common issues across countries, which likely makes our findings of interest to many countries. The focus on Austria can also be a strength since it homogenizes the results and allows for comparisons between different countries.

We used open-ended questions to collect information about barriers and facilitators for the implementation of EBP. Other qualitative research techniques, such as interviews or focus groups, might have offered us even more in-depth insights; however, they would not have been feasible in such a large sample. Therefore, we are confident that our approach helped us identify the most important barriers and facilitators among Austrian psychotherapists, with the additional benefit that the results are more representative because they are based on a large sample.

## Conclusions

Our study highlights that EBP is still not very popular within the psychotherapy community in Austria. Soft skills such as experience, competence, and the personality of the psychotherapist are perceived as more relevant to successfully treating patients than following an evidence-based approach. The combination with other perceived barriers such as organizational constraints and lack of knowledge/training in EBP methods may currently still prevent the broad application of EBP in Austria. The academization of psychotherapy training might change this in the future.

## Data Availability

The datasets analyzed during the current study are available from the corresponding author on reasonable request.

## Abbreviations

ANOVA: Analysis of variance
B: Behavioral
CHERRIS: CHEcklist for reporting results of internet surveys
EBP: Evidence-based practice
EBPAS-36: Evidence-based practice attitude scale-36
H: Humanistic
M: Mean
MD: Mean difference
n: Number of respondents
*p*: *P*-value
P: Psychodynamic
S: Systemic
SD: Standard deviation
