# Author Correction: A web-survey assessed attitudes toward evidence-based practice among psychotherapists in Austria

**DOI:** 10.1038/s41598-022-17572-7

**Published:** 2022-08-11

**Authors:** B. Nussbaumer-Streit, A. Jesser, E. Humer, A. Barke, B. K. Doering, B. Haid, W. Schimböck, A. Reisinger, M. Gasser, H. Eichberger-Heckmann, P. Stippl, G. Gartlehner, C. Pieh, T. Probst

**Affiliations:** 1 Department for Evidence-Based Medicine and Evaluation, University for Continuing Education Krems, Dr.-Karl-Dorrek Strasse 30, 3500 Krems a.d. Donau, Austria; 2 Department for Psychotherapy and Biopsychosocial Health, University for Continuing Education Krems, 3500 Krems, Austria; 3 Clinical and Biological Psychology, Catholic University of Eichstätt-Ingolstadt, 85072 Eichstätt, Germany; 4 Brandenburg Medical School Theodor Fontane, Neuruppin, Germany; 5 Austrian Federal Association for Psychotherapy, 1030 Vienna, Austria; 6 ABILE-Viktor Frankl Education Austria, 3390 Melk, Austria; 7 PROGES, 4020 Linz, Austria; 8 RTI International, 3400 Cornwallis Rd, Research Triangle Park, NC USA

Correction to: *Scientific Reports* 10.1038/s41598-022-13266-2, published online 07 June 2022

The original version of this Article contained an error in Table 4, where the asterisks were omitted for ”Years of experience”. The correct and incorrect value appears below.

Incorrect:

**Table:** 

Years of experience	− 0.111	*p* = 0.100	− 0.079	*p* = 0.241

Correct:

**Table:** 

Years of experience****	− 0.111	*p* = 0.100	− 0.079	*p* = 0.241

The original Article has been corrected.

